# Acute groin pain in pregnancy: a case of round ligament varicocele

**DOI:** 10.1259/bjrcr.20150517

**Published:** 2017-03-24

**Authors:** Soumya Cicilet

**Affiliations:** Department of Radiodiagnosis, St Johns Medical College, Bangalore, India

## Abstract

Varicocities of the round ligament is a relatively uncommon condition. Almost all the cases in literature had been reported in pregnant patients. It presents as a unilateral or bilateral painful or painless inguinal mass. Clinically it is difficult to distinguish from inguinal hernia on physical examination. We present a 22-year-old primigravida at 30 weeks of gestation with unilateral round ligament varicocele diagnosed on sonography and Doppler, who was managed conservatively.

## Background

Varicocities of the round ligament is a relatively uncommon condition.^1^ This entity presents as a unilateral or bilateral inguinal mass with or without pain.^2^ Almost all the cases in literature were in pregnant patients with one exception.^3,4^ In English literature 26 such cases have been reported till 2014.^4^ This is difficult to distinguish from inguinal hernia on physical examination.^5^ Hence when the swelling is painful the clinical suspicion of a strangulated inguinal hernia is high. This diagnostic dilemma raises the question of surgical management and the risk it poses to the pregnancy. Sonography and Doppler is a one stop shop answer to the question of management.^1,2,6^ We present a 22-year-old primigravida at 30 weeks of gestation with unilateral round ligament varicocele diagnosed on sonography with Doppler, who was managed conservatively.

## Case report

A 22-year-old primigravida at 30 weeks of gestation presented with a painful right inguinal swelling. Pregnancy had been uneventful. Symptoms started at 24 weeks when she noticed a swelling in the right groin which became prominent on standing and disappeared on lying down. On routine obstetric visit, her obstetrician made a provisional diagnosis of inguinal hernia and referred her to surgery department for further management. The surgeon also came to the same clinical diagnosis. As the swelling was not reducible he referred her to the department of radiology for an inguinal sonogram to rule out strangulation.

On physical examination there was a 4 × 2 cm soft tender mass in the right groin following the course of the inguinal canal which became prominent on valsalva and was not spontaneously reducing on supination.

**Figure 1.**
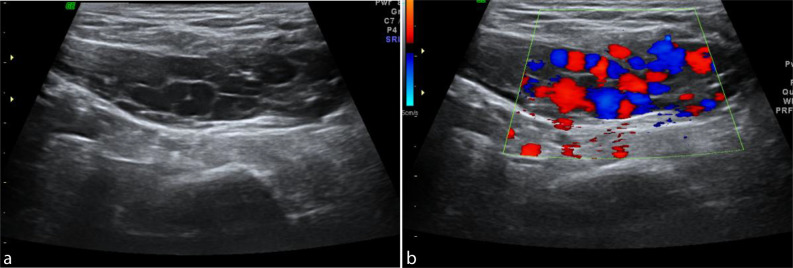
(a) Grey-scale sonogram along the longitudinal plane showing tubular tortuous anechoic channels in the right inguinal region. (b) Colour Doppler image showing flow signals in the vascular channels.

**Figure 2.**
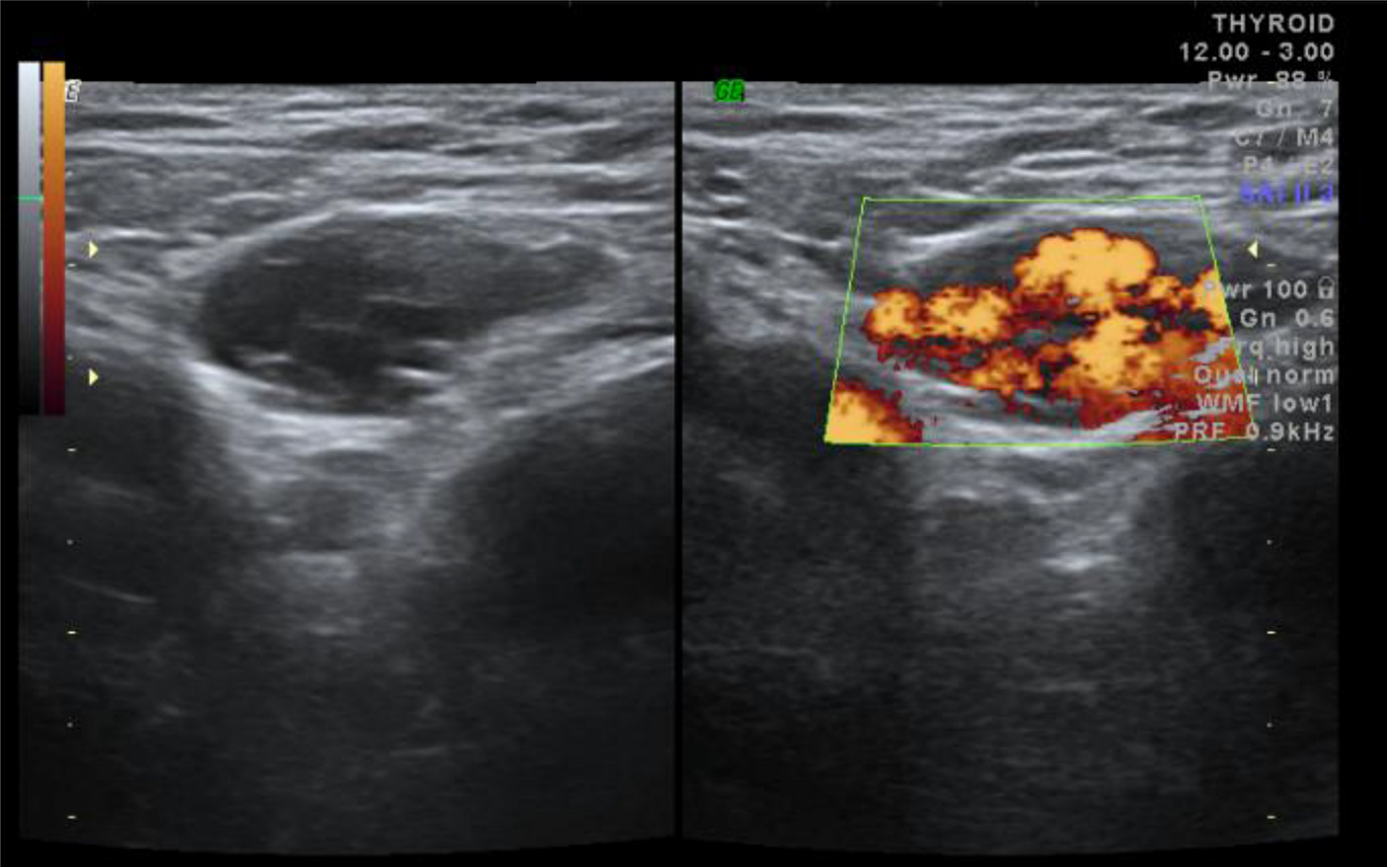
Transverse gray scale (left) and power Doppler (right) image in the right inguinal region showing vascular channels with flow signals on power Doppler.

**Figure 3.**
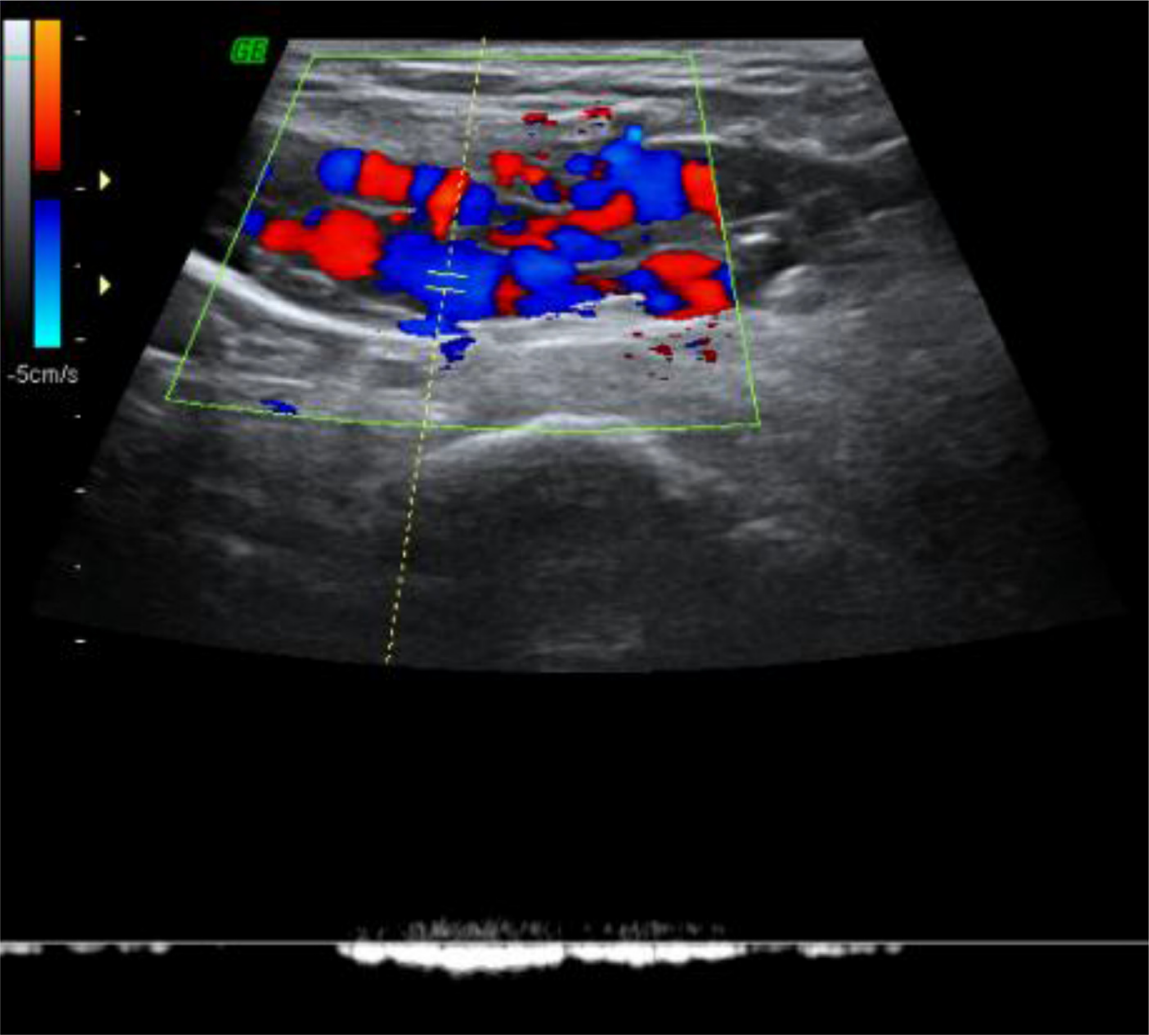
Pulse wave Doppler showing venous flow signal with reversal of flow on valsalva.

On grey-scale sonography performed using a 14 MHz linear transducer (Voluson 730 expert, GE medical systems, OH, USA) multiple anechoic tortuous tubular compressible channels were noted in the region of palpable mass (Figure 1a). Colour and power Doppler showed vascularity within these tubular channels (Figure 1b and Figure 2). Pulse wave Doppler confirmed venous flow with reversal on valsalva^7^ (Figure 3). As the vascular channels were completely compressible, echo free and showing complete filling on colour Doppler, the possibility of thrombosis was ruled out.^8^ There was no evidence of any herniating fat, bowel loop or lymphadenopathy in the inguinal region.^9^ The aforementioned sonological imaging findings were compatible with the diagnosis of round ligament varicocele.

The patient was managed conservatively and was followed up with repeated ultrasound evaluations on a monthly basis till term to rule out complications as she continued to have swelling and mild pain which was not increasing in intensity. She did not develop any complications and had an uneventful vaginal delivery at 38 weeks. The symptoms resolved completely by 3rd week postpartum.

## Discussion

Round ligament varicocele is an uncommon cause of inguinal pain. The true incidence of round ligament varicocele is not clearly mentioned in literature.^5^ All the cases reported in literature occurred during pregnancy except one.^4^ Mckenna et al^1^ in 2008 reported an incidence of five cases in 3816 deliveries. Kyeong Hwa Ryu, et al in 2014 summarized the clinicoradiological findings of 26 cases reported till then in English literature.^4^ Most commonly this condition is unilateral, more so on right side; however in 30% cases it is bilateral.^9^ In our case it was present only on right side.

The round ligament extends from the lateral angle of the uterus till the deep inguinal ring in the anterior leaf of the broad ligament and then courses through the inguinal canal till the labium majus. Veins in the round ligament drain into the inferior epigastric vein.^10^ Many factors contribute to the development of varicosities in the round ligament. They are venous obstruction due to mass effect caused by the gravid uterus, progesterone-mediated smooth muscle relaxation, increased cardiac output and increased venous return producing venous dilatation.^2,3^

On physical examination round ligament varicocele presents as an inguinal swelling with or without pain. This can be easily mistaken for an inguinal hernia as both present as inguinal masses with augmentation on valsalva or upright position and spontaneous reduction with supination.^4^ Once pain develops in the swelling there is high clinical suspicion of obstructed or strangulated hernia.

Accurate diagnosis of round ligament varicocele can be made using Doppler sonography.^2^ On grey-scale sonography dilated tortuous tubular channels are noted in the inguinal region with a “bag of worms” appearance which are completely compressible.^3^ On colour and pulse wave Doppler venous flow signals are noted within these channels.^1^ All the above-mentioned findings were seen in our case. Valsalva manoeuvre helps in identifying areas of slow flow at rest and even shows reversal of flow on pulse wave Doppler. The dilated veins can extend all the way into the parametrium and may coexist with pelvic venous congestion or lower limb varicose veins.^3^ Neither of these conditions were observed in our patient.

Differential diagnosis for round ligament varicocele are inguinal hernia, lipoma, lymphadenopathy, endometriosis, lymphangioma, hydrocele of canal of Nuck, abscess or hematoma.^1,2,7^ Inguinal hernia, even though is the commonest cause of inguinal mass during pregnancy, is relatively rare and has a reported incidence of 1 in 1000–3000 pregnant women.^6^ Ultrasonography with Doppler helps in ruling out all the above-mentioned entities as they have characteristic imaging findings and also helps in avoiding unwarranted surgery in an expectant patient.

As round ligament varicocele is a not an emergent condition and as it resolves spontaneously, conservative management is the rule.^1,4^ However, close follow-up is required as there is possibility of thrombosis or rupture of the varicosities and in which case surgical management may be warranted. Sonography also helps in identifying the complications. Absence of colour flow on Doppler, echogenic contents within the vascular channels and absence of compressibility are sonological signs of thrombosis.^4,9^ Our patient had no complications.

Round ligament varicocele is a rare but important cause of groin pain in pregnancy. Doppler sonography is an important diagnostic tool in determining the cause of acute groin pain in pregnant women. The correct diagnosis based on imaging makes a significant impact on management strategy as round ligament varicocele is managed conservatively.

## Learning points

1. Round ligament varicocele is an important cause of unilateral/bilateral inguinal mass with or without pain during pregnancy.
2. Doppler sonography is a one stop shop for diagnosing cause of groin swelling in pregnancy.
3. Sonography helps in avoiding unnecessary surgery in a pregnant patient by ruling out inguinal hernia.

## Consent

Written informed consent for the case to be published (including images, case history and data) was obtained from the patient(s) for publication of this case report, including accompanying images. 
